# Nanoparticles to Improve the Efficacy of Vaccines

**DOI:** 10.3390/pharmaceutics12050418

**Published:** 2020-05-02

**Authors:** Cheol-Heui Yun, Chong-Su Cho

**Affiliations:** Department of Agricultural Biotechnology and Research Institute of Agriculture and Life Sciences, College of Agriculture and Life Sciences, Seoul National University, 1 Gwanangno, Gwanak-gu, Seoul 08826, Korea

## Abstract

This editorial aims to summarize the nine scientific papers that contributed to the Special Issue entitled ‘Nanoparticles to Improve the Efficacy of Vaccines’.

A vaccine, providing active and protective immunity against a target disease, contains an agent that originated from and/or resembles a disease-causing microorganism. It is often made from a weakened or inactivated microbe, its toxins, or one of its nucleotides, peptides or proteins. Vaccines can be prophylactic to ameliorate or better prevent the effects of a wild-type pathogen, or therapeutic against likely cancers. To date, the World Health Organization lists twenty-seven preventable infections for which vaccines are available [1]—far less than what our society needs.

Vaccination, a process of introducing foreign antigenic material(s) in order to activate a host immune system, has been a key strategy to control diseases and improve quality of life in humans and animals. Despite the presence of some successful vaccines, many novel and modified diseases including Ebola virus disease, Zika virus disease, coronavirus diseases [middle eastern respiratory syndrome coronavirus (MERS-CoV), severe acute respiratory syndrome coronavirus (SARS-CoV), and 2019 novel coronavirus (2019-nCoV)], dengue fever, Marburg disease, malaria, and tuberculosis are in need of effective vaccines together with qualified adjuvants. While traditional adjuvants such as alum have been exclusively employed clinically to promote humoral responses, recent developments in adjuvant research have identified molecules, which are pathogen-associated molecular patterns, a few chemical compounds, and agonists of toll-like receptors, all of which induce strong immune responses.

With great advancements in the area of material science, a new era of innovative strategies for vaccine design has arrived, enabling the precise delivery of vaccines, the enhanced role of vaccine adjuvants, an increase in the sparing effect, better stabilization, and slow release at the induction site. Nanomaterials that modified to trigger antigen-specific immune responses could be categorized into liposomes and lipid-based nanoparticles, polymeric nanoparticles, gold nanoparticles, inorganic nanoparticles, virus-like particles, self-assembled proteins, and carbon-based nanoparticles (carbon nanotubes and graphenes). In the Special Issue, entitled ‘Nanoparticles to Improve the Efficacy of Vaccine’ in the Pharmaceutics (https://www.mdpi.com/journal/pharmaceutics/special_issues/Nanoparticles_Vaccines), we draw attention to the advanced platforms and technologies using nanomaterials in order to produce the best outcomes in terms of vaccination and immunological memory.

Kim et al. [2] described a method to improve the battle against intracellular bacterial or viral infections, and malignant tumors by various vaccination schemes, using physicochemical characteristics that target antigen-presenting cells (APCs). In particular, the improvement of an unconventional type of antigen presentation, called ‘cross-presentation’ in APCs when treated with certain nanomaterials for antigen-specific CD8+ T cell responses, was discussed [3]. The authors focused on the mechanisms of two major intracellular pathways that nano-sized vaccines harness for cross-presentation, namely endosomal swelling and rupture, and membrane fusion. These processes allow exogenous antigens exported from phagosomes into the cytosol, followed by loading on major histocompatibility complex (MHC) class I, triggering the clonal expansion of antigen-specific CD8+ T cells. 

Barnowski et al. [4] discussed nano-vaccines in the form of virus-like particles (VLPs), which share structural identities with their wild-type viruses, allowing B cells to face the natural conformation of the virus. The authors concentrated on the use of flagellin, a potent inducer of innate immunity via toll-like receptor 5, as an adjuvant to formulate human immunodeficiency virus (HIV)-based nanoparticle B cell-targeting vaccines that display either the HIV-1 envelope protein (Env) or a model antigen, hen egg lysozyme (HEL). They postulated that, in the context of VLP-based B cell nano-vaccines, flagellin may outcompete against a less immunogenic antigen, while, in combination with a strong immunogen, the adjuvanticity of flagellin dominates over its immunogenicity.

Kang et al. [5] introduced a VLP vaccine containing Rhoptry organelle proteins (ROP)4 and/or ROP13 secreted by *Toxoplasma gondii* together with influenza M1. It was intriguing that upon challenge via the oral route, mice immunized with ROP(4 + 13) VLPs elicited higher levels of *T. gondii*-specific IgG and IgA responses in fecal, urine, intestine and vaginal samples in accordance with CD4+ T, CD8+ T cells, and germinal center B cell responses when compared with other vaccines, ROP4 VLPs, ROP13 VLPs, and ROP4+ROP13 VLPs. Thus, the article shed light on important insights and potential strategies for the design of a vaccine using the *T. gondii* ROPs and VLP system.

Viyayan et al. [6] discussed biomimetic nanoparticles (NPs) to deliver vaccines for the treatment of diseases including HIV, malaria, some tumors and bacterial diseases due to their beneficial advantages such as improved antigen stability, targeted delivery, long-time controlled release and evasion of immune responses. They covered four kinds of biomimetic NPs for the delivery of vaccines. The first was liposomes, obtained by the dispersion of phospholipids in water, because they display high antigen loading and co-delivery of both hydrophobic and hydrophilic antigens. The second was NPs coated with cell membranes from red blood cells, leukocytes, cytotoxic T-cells, NK cells, platelets, macrophages or cancer cells, because they preserve the physicochemical properties of the core synthetic NPs and maintain the cellular composition on their membrane shell. The third was self-assembled proteins, because they play diverse physiological roles and mimic natural microbe architectures. The fourth was virus-like NPs containing noninfectious subsets of viruses, because they assemble without containing any viral RNA. 

Sartorius et al. [7] described the possibility of filamentous bacteriophages (FBs), prepared by phage display technology, as nature-made NPs to deliver therapeutic vaccines, because lytic bacteriophages have been used as antibacterial materials in several clinical trials due to their safe and inexpensive therapeutic behavior, where the FBs enter the blood vessels easily, owing to their nano size, and exhibit high-density antigen expression. The authors showed the induction of specific cytotoxic T cell responses to HLA-A2-restricted and hepatitis B virus epitopes due to the ability of the filamentous rod, internalized into APCs. It seems likely that the targeted delivery of these NPs in new-generation vaccines against tumors such as melanoma and mastocytoma might be realized, although clinical trials are necessary to establish their safety in humans. 

Lim et al. [8] described nano delivery systems for the improvement of the enhanced gene expression of DNA vaccines that provide potent humoral and cell-mediated responses. They pointed out the limitations of their clinical applications, which present hurdles in their delivery to targeted immune cells, although none have been approved for clinical use thus far. The authors discussed the advantages and weaknesses of polymeric NPs, lipid NPs, hybrid lipid-polymer NPs, inorganic NPs, virus-like NPs and protein-based NPs in the delivery of DNA vaccines. DNA vaccine platform technology would have a high chance to be successful in clinical application if the better adjuvants and/or the encoded antigens were selected properly.

Tan et al. [9] discussed norovirus capsid protein-derived NPs and polymers obtained by homotypically and/or heterotypically self-assembled mechanisms through bioengineering norovirus capsid shell (S) and protruding (P) domains, making them efficient vaccine candidates against noroviruses because they are easily produced, highly stable, they elicit strong host immune responses and several chimeric $S_{60}$ and $P_{24}$ NPs as well as P domain-derived polymers, as shown in other virus vaccine candidates such as rotavirus, influenza virus, EV 71, HIV-1, Alzheimer, and astrovirus diseases. It may be expected that $P_{24}$ NPs and polymers function as multifunctional P domain-based oligomer vaccine platforms displaying foreign antigens. 

There are two articles describing the importance of adjuvants. Park et al. [10] described a novel adjuvant in the form of a single-stranded RNA (ssRNA) nano-structure that can stimulate both Th1 and Th2 responses. The authors claimed that neither adverse side effects such as hematological and serum biochemical parameters, nor IgE and anti-nuclear antibodies, which are markers of autoimmune disease, was found, and postulated that this ssRNA nanostructure may be an alternative choice over traditional adjuvants. Sharma et al. [11] discussed the possibility of zinc oxide (ZnO)-based nanocomposites as vaccine adjuvants and the application of cancer immunotherapeutics because of their biocompatibility, unique physicochemical properties, cost-effective mass production, intrinsic adjuvant-like properties and immunomodulatory function. The authors introduced the effects of diverse formulations and compositions on the efficiency of antigen delivery and immunogenicity by augmenting antigen processing, enhancing antigen stability and controlling the release of antigens. They described that the immunomodulatory effects of these innate immune cells were mediated by intracellular ${\mathrm{Zn}}^{2+}$ dissolution in the lysosomes after the phagocytosis of particles and the generation of reactive oxygen species, resulting in the release of inflammatory cytokines and the activation of immune cells. Furthermore, they discussed the possibility of the enhancement of anti-cancer immunity when combined with tumor-specific antigens into ZnO NPs, although the potential toxicity and biodistribution of the ZnO NPs during in vivo studies should be thoroughly verified.

As vaccine development pushes toward less immunogenic components such as nucleotide- based, peptide-based or sub-unit vaccines because of their side effects and the life-threatening risks of live attenuated vaccines, strategies to boost both innate and adaptive immune responses are increasingly needed. In the present Special Issue, the authors discuss the modification of nanomaterials to provide a functional and stable interface for different applications and strategies for vaccination. We hope this Special Issue, highlighting the importance and use of nanomaterials as a platform technology, will direct scientists as well as manufacturers to improve the efficacy of vaccines that are currently under development against modified or novel diseases. 
